# Functional role of miR-10b in tamoxifen resistance of ER-positive breast cancer cells through down-regulation of HDAC4

**DOI:** 10.1186/s12885-015-1561-x

**Published:** 2015-07-24

**Authors:** Aamir Ahmad, Kevin R. Ginnebaugh, Shuping Yin, Aliccia Bollig-Fischer, Kaladhar B. Reddy, Fazlul H. Sarkar

**Affiliations:** 1 Department of Pathology, Karmanos Cancer Institute, Wayne State University School of Medicine, 740 HWCRC Bldg, 4100 John R. Street, Detroit, MI 48201 USA; 2 Department of Oncology, Karmanos Cancer Institute, Wayne State University School of Medicine, 740 HWCRC Bldg, 4100 John R. Street, Detroit, MI 48201 USA

**Keywords:** Tamoxifen resistance, miR-10b, HDAC4, ER-positive breast cancers

## Abstract

**Background:**

For breast cancer patients diagnosed with estrogen receptor (ER)-positive tumors, treatment with tamoxifen is the gold standard. A significant number of patients, however, develop resistance to tamoxifen, and management of such tamoxifen-resistant patients is a major clinical challenge. With an eye to identify novel targets for the treatment of tamoxifen-resistant tumors, we observed that tamoxifen-resistant cells derived from ER-positive MCF-7 cells (MCF7TR) exhibit an increased expression of microRNA-10b (miR-10b). A role of miR-10b in drug-resistance of breast cancer cells has never been investigated, although its is very well known to influence invasion and metastasis.

**Methods:**

To dileneate a role of miR-10b in tamoxifen-resistance, we over-expressed miR-10b in MCF-7 cells and down-regulated its levels in MCF7TR cells. The mechanistic role of HDAC4 in miR-10b-mediated tamoxifen resistance was studied using HDAC4 cDNA and HDAC4-specific siRNA in appropriate models.

**Results:**

Over-expression of miR-10b in ER-positive MCF-7 and T47D cells led to increased resistance to tamoxifen and an attenuation of tamoxifen-mediated inhibition of migration, whereas down-regulation of miR-10b in MCF7TR cells resulted in increased sensitivity to tamoxifen. Luciferase assays identified HDAC4 as a direct target of miR-10b. In MCF7TR cells, we observed down-regulation of HDAC4 by miR-10b. HDAC4-specific siRNA-mediated inactivation of HDAC4 in MCF-7 cells led to acquisition of tamoxifen resistance, and, moreover, reduction of HDAC4 in MCF7TR cells by HDAC4-specific siRNA transfection resulted in further enhancement of tamoxifen-resistance.

**Conclusions:**

We propose miR-10b-HDAC4 nexus as one of the molecular mechanism of tamoxifen resistance which can potentially be expolited as a novel targeted therapeutic approach for the clinical management of tamoxifen-resistant breast cancers.

**Electronic supplementary material:**

The online version of this article (doi:10.1186/s12885-015-1561-x) contains supplementary material, which is available to authorized users.

## Background

The problem of drug-resistance is a major clinical concern for the successful management of cancer patients. Estrogen receptor (ER) is expressed in 75 % of breast cancers [1] and for such breast cancers, tamoxifen is one of the important drug of choice for targeted personalized therapy. Tamoxifen can significantly lower the chances of developing recurrent breast cancer and can be very effective in women who initially present with metastatic disease. It remains the primary therapeutic agent for the management of ER and/or progesterone receptor (PR)-expressing breast cancers, particularly in premenopausal women without or with conventional chemotherapeutics. However, many ER-positive cancers that initially respond to tamoxifen, eventually develop resistance with the continued administration of the drug [2]. Acquired resistance to tamoxifen is seen in 30–40 % of breast cancer patients treated with tamoxifen for 5 years [3], which clearly indicates that this is a major clinical problem. The tumors that have acquired drug resistance are usually far more aggressive and difficult to treat with conventional therapeutics. They are invariably linked to poor prognosis as well as overall poor survival.

There is an emerging interest in microRNAs (miRNAs) as therapeutic targets in drug-resistant cancers [4]. These short non-coding RNAs have been implicated in multiple stages of cancer progression and metastasis, and reports in the last few years have indicated the involvement of miRNAs in tamoxifen resistance as well [5–11]. The miRNAs directly or indirectly implicated in tamoxifen resistance in breast cancer models include miR-221/222 [5, 6], miR-15a/16 [7], miR-342 [8], miR-375 [9], miR-200 s [10], miR-126/miR-10a [11] and miR-519a [12]. We designed the current study to investigate miRNA-regulation of tamoxifen resistance, and used paired cell lines – parental MCF-7 and tamoxifen resistant MCF-7 (MCF7TR) as our model. Tamoxifen resistance has been linked to epithelial-mesenchymal transition (EMT) through an involvement of miR-375 [9], and EMT-regulating miRNAs such as miR-200 s [13, 14] and let7s [15] have been reported to play a role in resistance to tamoxifen [10]. In our model, we observed increased invasion of MCF7TR cells, a phenomenon which has been linked to EMT [16], which prompted us to investigate the miRNAs that have been linked to invasion and EMT characteristics of breast cancer cells. We observed a significant over-expression of miR-10b in MCF7TR cells which correlated with acquired tamoxifen resistance. Mechanistically, we identified HDAC4 as a target of miR-10b which mediated the miR-10b action. Our results provide the first evidence in support of such action of miR-10b and HDAC4 and further highlight the importance of miRNA-regulation in drug resistance phenotype.

## Methods

### Cell lines and reagents

MCF-7 and T47D breast cancer cells were purchased from ATCC and maintained in DMEM and RPMI mediam (Invitrogen, Carlsbad, CA), respectively, with 10 % fetal bovine serum, 100 units/ml penicillin, and 100 μg/ml streptomycin in a 5 % CO_2_ atmosphere at 37 °C. The tamoxifen resistant MCF-7 derivatives, MCFTR cells, were generated by culturing parental MCF-7 cells in DMEM medium supplemented with 5 % FBS, antibiotics and 10^−6^ M 4-hydroxy tamoxifen. Concentration of tamoxifen was gradually increased until the final concentration was 10^−6^ M. Cells were continuously exposed to tamoxifen for 6 months during which time the medium was replaced every 3 to 4 days. The cell lines have been tested and authenticated in the core facility (Applied Genomics Technology Center at Wayne State University) by short tandem repeat profiling using the PowerPlex 16 System from Promega. Antibodies were purchased from following sources – HDAC4 (Cell Signaling) and β-actin (Sigma-Aldrich).

### Western blot analysis

For Western blot analysis, cells were lysed in RIPA buffer containing complete mini EDTA-free protease inhibitor cocktail (Roche) and phosphatase inhibitor cocktails 1 and 2 (Sigma-Aldrich). After resolution on 12 % polyacrylamide gels under denaturing conditions, proteins were transferred to nitrocellulose membranes, incubated with appropriate primary/horseradish peroxidase-conjugated secondary antibodies and visualized using chemiluminescence detection system (Pierce).

### Cell growth inhibition studies by 3-(4,5-dimethylthiazol-2-yl)-2,5-diphenyltetrazolium bromide (MTT) assay

Cells were seeded at a density of 5 x 10^3^ cells per well in 96-well culture plates. After overnight incubation, liquid medium was removed and replaced with a fresh medium containing DMSO (vehicle control) or different concentrations of tamoxifen, as indicated. After 48 h, 25 μl of 3-(4,5-dimethylthiazol-2-yl)-2,5-diphenyltetrazolium bromide (MTT) solution (5 mg/ml in phosphate-buffered saline, PBS) was added to each well and incubated further for 2 h at 37 °C. Upon termination, the supernatant was aspirated and the MTT formazan, formed by metabolically viable cells, was dissolved in DMSO (100 μl) by mixing for 30 min on a gyratory shaker. The absorbance was measured at 595 nm on Ultra Multifunctional Microplate Reader (TECAN, Durham, NC).

### Cell viability studies by Trypan Blue assay

Cells were seeded in 6-well culture plates and appropriately treated. Upon completion of incubation, culture medium (with floating dead cells) was collected and pooled with the adherent cells removed from the plate by trypsinization. The cells were briefly spun and re-suspended in the normal culture medium. Cell viability was assessed by adding 50 μl of Trypan Blue solution (0.4 % in PBS) to 50 μl of the cell suspension. After 2 min, the number of living cells, which did not retain the dye was counted using a hemocytometer, and was compared to the total number of cells (living + dead) to calculate the viability percentage.

### Histone/DNA ELISA for detection of apoptosis

The Cell Death Detection ELISA Kit (Roche) was used to detect apoptosis. Cells were treated, as indicated for individual experiments. After treatment, the cytoplasmic histone/DNA fragments from these cells were extracted and incubated in the microtiter plate modules coated with anti-histone antibody. Subsequently, the peroxidase-conjugated anti-DNA antibody was used for the detection of immobilized histone/DNA fragments followed by color development with ABTS substrate for peroxidase. The spectrophotometric absorbance of the samples was determined by using Ultra Multifunctional Microplate Reader (TECAN) at 405 nm.

### miRNA transfections

Transfections of pre/anti-miR-10b were done using methodology previously described [13]. Briefly, cells were seeded (2.5 × 10^5^ cells per well) in six well plates and transfected with pre/anti-miR-10b or non-specific pre/anti-miRNA controls (Life Technologies) at a final concentration of 200 nM, using DharmaFECT transfection reagent (Dharmacon). After 48 h of transfection, cells were passaged and transfected once again before being used in the experiment.

### Real-time RT-PCR

Real-Time RT-PCR analyses were done as described previously [13]. Total RNA was isolated using the mirVana miRNA isolation kit (Life Technologies). The levels of miRNAs were determined using miRNA-specific Taqman probes from the Taqman MicroRNA Assay (Life Technologies). The relative amounts of miRNA were normalized to RNU48.

### Cell migration and invasion assays

Cell migration and invasion assays were performed using 24 well transwell permeable supports with 8 μM pores (Corning) [13]. After transfections with pre/anti-miR-10b or the non-specific controls, as described above, cells were suspended in serum free medium and seeded into the transwell inserts. For invasion assays, the transwell inserts were coated with growth factor reduced Matrigel (BD Biosciences). Bottom wells were filled with complete media. After 24 h, cells were stained with 4 μg/ml calcein AM (Life Technologies) in PBS at 37 °C for 1 h. Cells were detached from inserts by trypsinization and fluorescence of the invaded cells was read in ULTRA Multifunctional Microplate Reader (TECAN, San Jose, CA).

### Luciferase assay

For luciferase reporter assays, MCF-7 cells were co-transfected with HDAC4 3′UTR luciferase vector (GeneCopoeia, Catalog # HmiT023167-MT05) and pre-miR-10b or miRNA negative control, using DharmaFECT Duo Transfection Reagent (Dharmacon). The vector has HDAC4 3′ UTR sequence inserted downstream of the secreted Gaussia luciferase (GLuc) reporter gene system, driven by SV40 promoter for expression in mammalian cells. A secreted Alkaline Phosphatase (SEAP) reporter, driven by a CMV promoter, is also cloned into the same vector (pEZX-MT05) and serves as the internal control. 48 h post- transfection, Gluc and SEAP luciferase activities were assayed using Secrete-Pair^™^Dual Luminescence Assay Kit (GeneCopoeia), following exactly the same procedure as described in the vendor’s protocol.

## Results

### Tamoxifen resistant MCF7 showed elevated expression of miR-10b

**Fig. 1:**
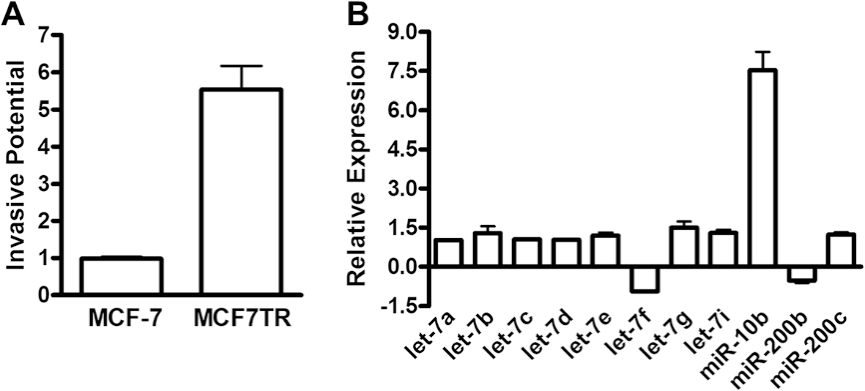
miR-10b in tamoxifen-resistant MCF-7 cells. **a** Tamoxifen-resistant MCF-7 cells (MCF7TR) showed significantly higher invasive potential, compared to parental MCF-7 cells. **b** Screening of miRNAs in MCF7TR cells, relative to the levels in MCF-7 cells, by real time RT-PCR. RNU48 was used as internal control for the real-time RT-PCR miRNA analysis.

Tamoxifen resistance has been linked with epithelial-to-mesenchymal transition (EMT) [9, 16], which in turn is linked to breast cancer invasion [16]. Therefore, we started our investigations with an evaluation of the relative invasive potential of MCF7TR cells, and we found that MCF7TR cells are highly invasive (*p* < 0.01), compared to their parental cells (Fig. 1a). Next we screened several miRNAs that have been linked with EMT and invasion of breast cancer cells, namely let-7 s, miR-200 s and miR-10b. No significant difference in the expression of let-7 and miR-200 family miRNAs was observed in MCF7TR cells, relative to MCF-7 cells; however, a very significant up-regulation (more than 7-folds, *p* < 0.01) of miR-10b expression was seen (Fig. 1b).

### miR-10b expression correlated with tamoxifen sensitivity

**Fig. 2:**
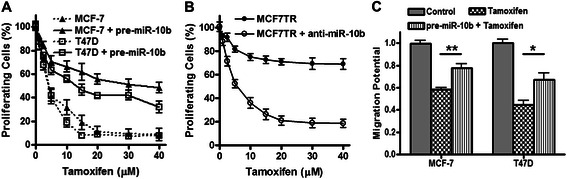
Effect of miR-10b levels on response to tamoxifen. **a** Ectopic over-expression of miR-10b in MCF-7 and T47D cells, through transfections with pre-miR-10b oligonucleotides, increased tamoxifen resistance, (**b**) silencing of miR-10b in MCF7TR cells, through transfections with anti-miR-10b oligonucleotides, decreased their tamoxifen resistance and (**c**) ectopic over-expression of miR-10b in MCF-7 and T47D cells significantly attenuated tamoxifen-induced inhibition of migration potential. Cells were treated with indicated doses of tamoxifen for 48 h. *p < 0.05, **p < 0.01

To investigate whether elevated miR-10b levels in MCF7TR cells may have a role in determining resistance to tamoxifen, we over-expressed miR-10b in parental MCF-7 cells and exposed the cells to increasing concentrations of tamoxifen. We observed a dose-dependent inhibition of cell growth in control MCF-7 cells with IC-50 less than 5 μM, and more than 90 % inhibition at 20 μM tamoxifen concentration (Fig. 2a). However, a significant resistance to tamoxifen was seen in MCF-7 cells that were transfected with pre-miR-10b, with IC-50 increased ~7-8-folds (Fig. 2a). In order to rule out cell line-specific effects, we confirmed our findings in another ER-positive breast cancer cell line, T47D. Similar to MCF-7 cells, tamoxifen inhibited cell proliferation in T47D cells but transfections with pre-miR-10b resulted in a significant inhibition of tamoxifen action in the T47D cells as well (Fig. 2a), which is similar to the data obtained from the MCF7 cells. Further, we confirmed our results in a reciprocal experimental setup where we down-regulated miR-10b in MCF7TR cells. As shown in Fig. 2b, we found that control MCF7TR cells are quite resistant to tamoxifen but antagonizing miR-10b expression, by the use of specific anti-miR-10b oligonucleotides, resulted in sensitization of these cells to tamoxifen with IC50 close to 5 μM. Next, we evaluated the effect of tamoxifen on migration potential of MCF-7 and T47D cells and observed a marked reduction in migration of both of these cell lines when treated at a dose of 5 μM for 48 h (Fig. 2c). However, prior transfections with miR-10b significantly (*p* < 0.001 for MCF-7 and *p* < 0.05 for T47D cells) attenuated the tamoxifen-mediated inhibition of migration (Fig. 2c). Since we observed similar results in both MCF-7 and T47D cells, we performed further mechanistic studies in MCF-7 cells.

**Fig. 3:**
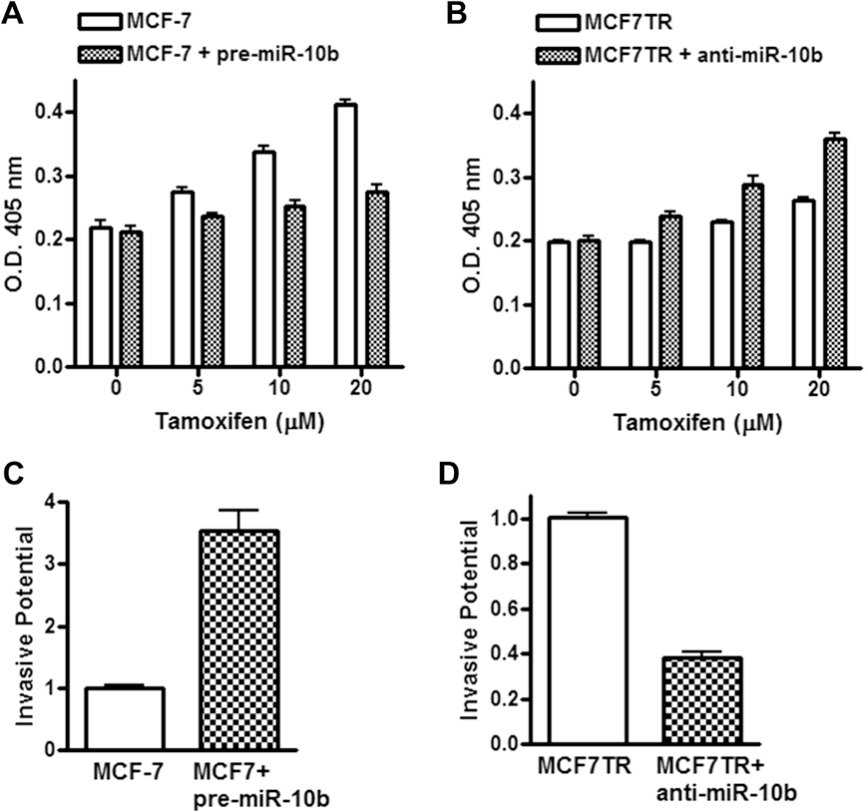
Effect of miR-10b levels on apoptosis-induction and invasion. Effect of miR-10b levels on apoptosis-induction in (**a**) MCF-7 and (**b**) MCF7TR cells. Induction of apoptosis was assessed by DNA Histone-ELISA assay. Invasion of (**c**) MCF-7 and (**d**) MCF7TR cells was assessed by plating cells in matrigel-coated plates *MCF-7,* non-specific pre-miRNAs transfected MCF-7 cells; *MCF-7 + pre-miR-10b,* pre-miR-10b transfected MCF-7 cells; *MCF7TR,* non-specific anti-miRNAs transfected MCF7TR cells; *MCF7TR + anti-miR-10b,* anti-miR-10b transfected MCF7TR cells.

It is known that tamoxifen induces apoptosis in MCF-7 cells [7, 8], therefore, we looked at the tamoxifen-induced apoptosis in our model system to further correlate miR-10b levels with tamoxifen action. Tamoxifen treatment resulted in the induction of apoptosis in a dose-dependent manner in MCF-7 cells which was attenuated by transfection with pre-miR-10b (Fig. 3a). In MCF7TR cells, while non-specific anti-miRNA transfected cells did not exhibit much induction of apoptosis, transfection of anti-miR-10b resulted in a dose-dependent induction of apoptosis (Fig. 3b), again suggestive of sensitization of these cells to tamoxifen through deregulation of miR-10b. We also looked at the effect of miR-10b expression on invasive potential. Pre-miR-10b transfected MCF-7 cells were significantly much more invasive (Fig. 3c) while anti-miR-10b transfected MCF7TR cells were significantly less invasive, compared to respective controls (Fig. 3d). Collectively, these results provided a clear functional involvement of miR-10b in tamoxifen resistance.

### HDAC4 is a novel target of miR-10b

**Fig. 4:**
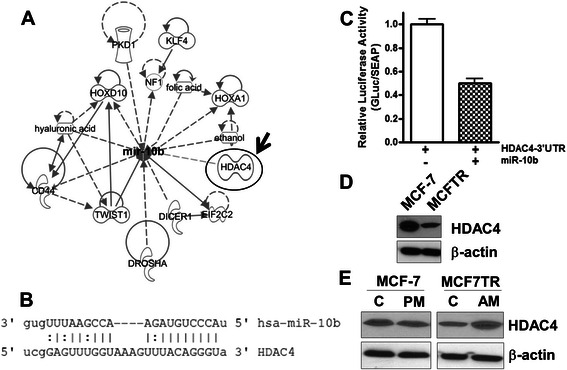
HDAC4 is a target of miR-10b. **a** Ingenuity Pathway Analysis for targets of miR-10b. HDAC4 is shown with an arrow. **b** Sequence complementarities of miR-10b and its target HDAC4. **c** Luciferase assay was conducted to confirm that HDAC4 is a direct target of miR-10b. MCF-7 cells were co-transfected with dual luciferase plasmid pEZX-MT05-HDAC4-3′UTR along with a control pre-miR or pre-miR-10b, and assayed for luciferase activity 48 h after transfection. **d** Levels of HDAC4 in parental (MCF-7) and tamoxifen-resistance MCF-7 (MCF7TR) cells. **e** Effect of altered miR-10b levels on HDAC4 levels. β-actin protein was used as protein loading control for Western blots and RNU48 was used as internal control for the real-time RT-PCR miRNA analysis. *C*, control; *PM*, pre-miR-10b; *AM*, anti-miR-10b

Having established a role of miR-10b in tamoxifen resistance, we next studied the molecular mechanism of such action of miR-10b by looking at its potential targets. We started with an Ingenuity Pathway Analysis to list the potential targets of miR-10b. A number of targets such as CD44, TWIST, HOXA1, HOXD10, HDAC4, PKD1, KLF4, etc. were found (Fig. 4a). We further scanned TargetScan/microRNA.org as well as reported literature for the potential targets of miR-10b and tested whether the potential targets were differentially expressed in MCR-7 vs. MCF7TR cells. Based on such screening, we focused on HDAC4, and the results presented in Fig. 4b show an alignment of miR-10b with its predicted site on HDAC4′s 3′ UTR. Next, we performed luciferase assays to confirm binding of miR-10b to 3′UTR of HDAC4. MCF-7 cells were co-transfected with pre-miR-10b (or control pre-miRNA) and pEZX-MT05 vector that carried the cloned HDAC4 3′UTR sequence. As can be seen in Fig. 4c, the luciferase activity was inhibited in cells transfected with pre-miR-10b by almost 50 %, compared to the control cells. These results suggested a direct binding of miR-10b to 3′UTR of HDAC4. Consistent with this direct evidence, we further obtained additional data in support of HDAC4 being a valid target of miR-10b, and we found significantly down-regulated expression of HDAC4 in high miR-10b expressing MCF7TR cells (Fig. 4d). To further establish the regulation of HDAC4 by miR-10b, we also tested the expression of HDAC4 in parental MCF-7 cells with or without transfection with pre-miR-10b. Ectopic expression of miR-10b resulted in the down-regulation of HDAC4 in MCF-7 cells and, conversely, down-regulation of miR-10b in MCF7TR cells resulted in increased expression of HDAC4 (Fig. 4e).

### HDAC4 is mechanistically involved in miR-10b-influenced tamoxifen resistance

**Fig. 5:**
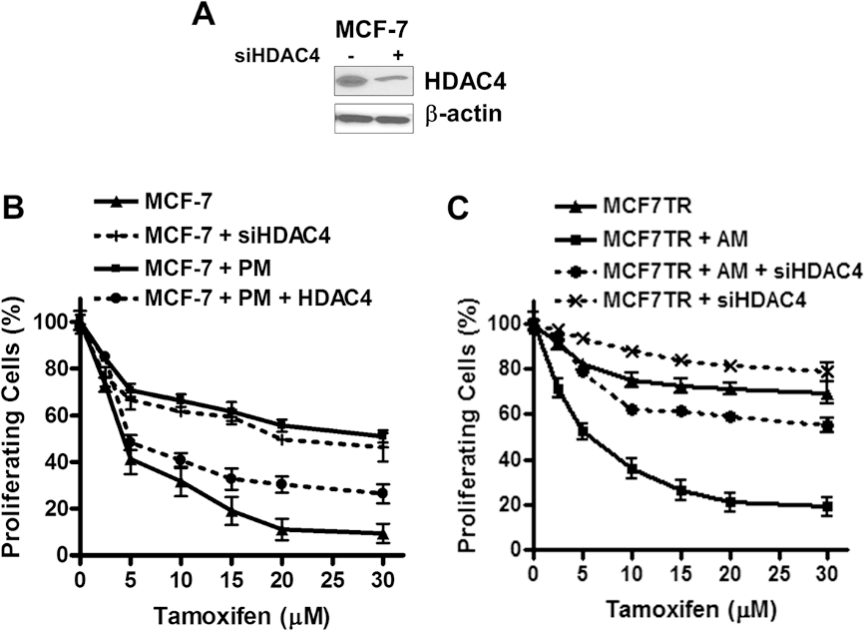
Effect of HDAC4 levels on tamoxifen-sensitivity. **a** siRNA against HDAC4 reduced its expression in MCF-7 cells. Functional role of HDAC4 and miR-10b on tamoxifen sensitivity in **b** MCF-7 and **c** MCF7TR cells. β-actin protein was used as protein loading control for Western blots. Tamoxifen treatment was done for 48 h at indicated doses. *PM*, pre-miR-10b; *AM*, anti-miR-10b; *HDAC4*, HDAC cDNA; *siHDAC4*, siRNA against HDAC4

Next we asked the question whether miR-10b mediated regulation of HDAC4 is relevant to miR-10b’s influence on tamoxifen resistance. We used specific siRNA against HDAC4 to down-regulate its expression. Fig. 5a demonstrates an efficient down-regulation of HDAC4 by siRNA. When exposed to increasing concentrations of tamoxifen, silencing of HDAC4 mimicked the effects of transfections with pre-miR-10b (Fig. 5b). Moreover, re-expression of HDAC4 in pre-miR-10b transfected MCF-7 cells, by the use of HDAC4 cDNA, re-sensitized these cells to tamoxifen. As a confirmation of our results in the reciprocal model, antagonizing miR-10b made MCF7TR cells responsive to tamoxifen, and silencing of HDAC4 in these very cells made the cells resistant to tamoxifen (Fig. 5c). Although MCF7TR cells already have low basal levels of HDAC4 (Fig. 4d), further knock-down of HDAC4 by the use of specific siRNA led to further diminishing the effects of increasing doses of tamoxifen (Fig. 5c).

### HDAC4 regulation by miR-10b determines cellular response to tamoxifen

**Fig. 6:**
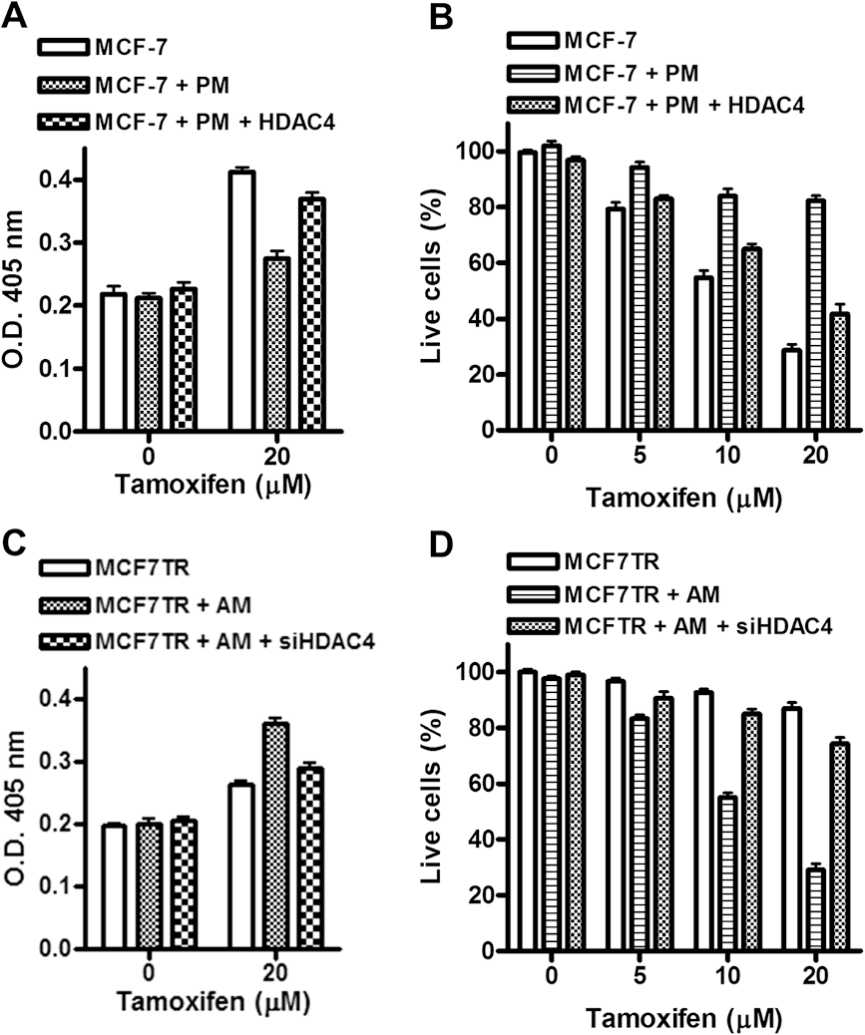
miR-10b and its target HDAC4 influence tamoxifen-induced apoptosis and cell viability. Effect of ectopic expression of miR-10b and HDAC4 on (**a**) apoptosis-induction and (**b**) viability of MCF-7 cells, and the effect of silencing of miR-10b and HDAC4 on (**c**) apoptosis-induction and (**d**) viability of MCF7TR cells. Tamoxifen treatment was for 48 h. *PM*, pre-miR-10b; *AM*, anti-miR-10b; *HDAC4*, HDAC cDNA; *siHDAC4*, siRNA against HDAC4

We subsequently tested the role of HDAC4 in tamoxifen-induced apoptosis and found that whereas pre-miR-10b transfection made MCF-7 cell resistant to tamoxifen-induced apoptosis, an effect particularly evident at 20 μM dose (Fig. 6a), re-expression of HDAC4 led to overcome tamoxifen resistance. A similar effect was seen when we quantitated live cells after tamoxifen treatment, and re-expression of HDAC4 clearly negated the effects of miR-10b transfection (Fig. 6b). In MCF7TR cells, silencing of HDAC4 was observed to reverse the effects of anti-miR-10b, both on apoptosis induction (Fig. 6c) as well as viability of cells (Fig. 6d).

Taken together, these results demonstrated a functional importance of HDAC4 in miR-10b-mediated response of ER-positive cells to tamoxifen treatment. Finally, we questioned whether there is any evidence for such molecular events in clinical samples. While there is evidence connecting miR-10b with clinical outcome in breast cancer patients [17], no such information is available for HDAC4. To evaluate if the down-regulation of HDAC4, as observed by us, has any clinical significance, we turned to public databases and data-mining tools. We first searched for evidence of under-expression of HDAC4 in breast cancer patients, relative to normal patients, using the online data mining platform Oncomine. We found a few studies/data sets supporting down-regulation of HDAC4 in breast cancer samples, relative to normal controls (*p* ≤ 0.05) (Additional file 1: Table S1). We also evaluated the correlation of HDAC4 expression with relapse free survival of breast cancer patients. For this, we turned to Kaplan Meier plotter, a publicly available tool that integrates survival data from GEO, EGA and TCGA to generate Kaplan Meier plots [18]. As seen in Additional file 2: Figure S1, breast cancer patients with low expression of HDAC4 had poor relapse free survival, compared to those with high expression (*p* < 0.01). A total of 3554 breast cancer samples were analysed for this comprehensive relapse free survival plot. We also turned to Oncomine database to look for clinical data on HDAC4 expression in ER-positive samples and observed lower expression of HDAC4 in ER-positive samples in, at least, one study (Additional file 3: Figure S2). Interestingly, there is evidence to suggest a negative correlation between HDAC4 and ER, where HDAC4 was found to transcriptionally suppress ER expression [19]. Collectively, the data mining from public databases supports our conclusions, suggesting that lower HDAC4 levels correlate with advanced breast cancers with poor prognosis.

## Discussion

The major conclusions from our present study are a) endogenous levels of miR-10b are significantly higher in MCF7TR cells, the tamoxifen-resistant derivatives of MCF-7 cells; b) induced expression of miR-10b in MCF-7 cells, by pre-miR-10b oligonucleotides, was correlated with increased invasion and resistance to tamoxifen-induced apoptosis while reduced expression of miR-10b in MCF7TR cells, by anti-miR-10b oligonucleotides, inhibited invasion along with reduced resistance to tamoxifen; c) HDAC4 appears to be an important target of miR-10b; its expression was found to correlate inversely with miR-10b levels and its levels modulated by altered miR-10b levels; and d) functional significance of HDAC4 regulation by miR-10b was suggested by the observation that over-expression of HDAC4 reversed tamoxifen resistance induced by ectopic expression of miR-10b in MCF cells, and silencing of HDAC4 attenuated the effects of anti-miR-10b transfections in MCF7TR cells.

Most targeted therapies are known to work initially but with the passage of time and continued administration, patients eventually develop resistance to the therapeutic agent, and this process is called extrinsic (acquired) drug resistance. While intrinsic (*de novo)* drug resistance characterized by resistance to therapy right from the beginning is itself clinically challenging, the phenomenon of acquired drug resistance is equally a big concern. Tamoxifen is an ER-targeting drug which is used for the successful management of ER-driven breast cancers. Acquired resistance to tamoxifen [20] is a major clinical concern and a survey of literature suggests that the major mechanisms currently under investigation include EMT and the cancer stem cells (CSCs). Multiple studies have provided direct as well as indirect evidence supporting this notion. In support of a mechanistic role of EMT in tamoxifen resistance of breast cancer cells, over-expression of Pin-1 [21], AKT [22], Nicastrin and Notch4 [23], FoxM1 [24], brachyury [25] as well as modulation of several microRNAs [9, 10] has been reported. Involvement of CSCs in tamoxifen resistance of breast cancer cells has been reported, which appears to be mechanistically linked with higher expression of CXCR4 [26], STAT3 [27], Sox2 [28], EZH2 [29], and lower expression of CD24 [30, 31].

Here we report a novel role of miR-10b in tamoxifen resistance of breast cancer cells. Tamoxifen-resistant breast cancer cells exhibit increased invasive potential, a phenomenon that is well established for high miR-10b expressing breast cancer cells [17, 32]. A recent report [33] has identified critical role of miR-10b in TGF-β1-induced EMT. In this work, miR-10b was found to be a downstream target of TGF-β1, essential for TGF-β1-induced down-regulation of epithelial marker E-cadherin and up-regulation of mesenchymal marker vimentin. Inhibition of miR-10b in metastatic breast cancer MDA-MB-231 and MDA-MB-435 cells significantly reversed the TGF-β1 effects. Further, a role of miR-10b in proliferation and growth of CSCs, *in vitro* as well as *in vivo*, has also been reported [34]. Thus, it appears that miR-10b is functionally involved in the induction of EMT and CSCs phenotypes, which would explain its role in drug resistance phenotype, such as tamoxifen resistance as observed in our study. While our work is the first report on mechanistic involvement of miR-10b in drug resistance of breast cancer cells, such role of miR-10b in other cancer models has been reported. miR-10b was observed to be consistently high in all the cisplatin resistant sublines derived from parental cisplatin-sensitive germ cell tumor cell lines [35], and it was reported to confer resistance to 5-fluorouracil in colorectal cancer cells [36]. Clearly, there is evidence in support of miR-10b-mediated induction of drug resistance which is in direct agreement with our findings.

The miRNA-mediated regulation of tamoxifen resistance has been studied in breast cancer models for many years where miR-221 and miR-222 are the most well characterized microRNAs [6, 5, 37, 38]. These oncogenic miRNAs confer resistance to tamoxifen through down-regulation of tumor suppressors p27Kip1 [6, 38] and TIMP3 [37]. Another oncogenic miRNA, miR-519a induces tamoxifen resistance via regulation of several tumor suppressor genes in PI3K pathway [12]. Not all miRNAs that are functionally involved in tamoxifen resistance are oncogenic. Tumor suppressors miR-15a and miR-16 regulate tamoxifen sensitivity by targeting Bcl-2 [7], miR-451 targets 14-3-3ζ [39], let7s target ER-α36 [15], miR-375 targets metadherin [9] and miR-200b/c target ZEB1 [10]. Also, an elevated expression of miR-126 and miR-10a has been linked to better prognosis and longer relapse-free time in breast cancer patients treated with tamoxifen [11]. Thus, the regulation of sensitivity to tamoxifen is influenced by both oncogenic and tumor suppressive miRNAs. Our results are suggestive of an oncogenic role of miR-10b. We used multiple bioinformatics-based methodologies to find a functionally viable target of miR-10b in our model system. Using IPA and online tools, we identified HDAC4 as a target of miR-10b, which was correlated with tamoxifen resistance/sensitivity, as determined by over-expression/silencing studies.

HDAC4 is a member of class IIa histone deacetylases and our results support an inverse relationship between HDAC4 expression and tamoxifen resistance. This is surprising, given the focus on HDAC inhibitors as anticancer agents. Consistent with the many reports on tumor-progressing role of HDACs, HDAC4 has been reported to be tumorigenic in different human cancers [40, 41]. Indicative of a tumor suppressor function of HDAC4 is the observation that HDAC4 was down-regulated in 15 of 18 urothelial cancer cell lines [42]. The paradox of HDAC4 activity also extends to its involvement in drug resistance. A number of reports present a positive correlation between HDAC4 expression and drug resistance. For instance, HDAC4 was shown to activate STAT1 leading to platinum resistance in ovarian cancer patients-derived cell lines [43] and resistance to etoposide in lung cancer cells [44]. HDAC4 also induced resistance to 5-fluorouracil in breast cancer cells [45] and inhibited docetaxel-related cytotoxicity in gastric cancer cells [46]. A careful review of the literature revealed that the only miRNA that has been associated with HDAC4, in the context of drug resistance, is miR-140 [47]. Interestingly, this study found a very similar function of HDAC4, as observed by us in the current study. Performed in colon and osteosarcoma cells, this study reported higher miR-140 expression in colon CSCs with increased resistance to 5-fluorouracil. HDAC4 inhibition was proposed as the mechanism of miR-140-induced chemoresistance. Thus, the only published work that investigated miRNA regulation of HDAC4 in resistant cells documented similar findings consistent with our results. In an earlier published work [48], we demonstrated that inhibitors of HDACs, such as Trichostatin A (TSA) and Suberoylanilide hydroxamic acid (SAHA), induced EMT in prostate cancer cells, as evidenced by up-regulated markers of mesenchymal phenotype. Further, TSA treatment resulted in increased expression of Sox2 and nonog indicating an enrichment of CSCs. Thus, antagonizing HDACs made the cancer cells more invasive, which is in agreement with our current findings, and, moreover, we provide here a mechanism through the novel involvement of miR-10b. It is tempting to suggest that such EMT/CSC-inducing activity of HDAC inhibitors might be a factor for their disappointing progress in clinical trials. Combined with the results from this study where low levels of HDAC4 correlated with drug resistance, it is important that the mechanistic involvement of HDACs in EMT, CSCs and drug resistance be evaluated in-depth before their selective targeting in clinics.

## Conclusions

The preliminary evidences supporting functional role of reduced HDAC4 in drug resistant cancer cells are available and more detailed studies need to be performed to further understand the complex relationship between microRNAs, HDACs, EMT and CSCs – all of which play important roles in determining response to conventional therapeutics. In recent years, the concept of personalized medicine has gained a lot of attention. The epigenetic approach for personalized medicine has largely focused on methyltransferases and the histone deacetylases. Towards this end, further characterization of the function of HDAC4 in drug resistance will be an important step forward towards realizing the goal of personalized medicine in the management of breast cancer patients, particularly those with recurrent disease.

## Additional files

Additional file 1: Table S1. Oncomine data supporting under-expression of HDAC4 in breast cancer samples, compared to normal controls. (DOCX 25 kb)
Additional file 2: Figure S1. Comparison of Relapse Free Survival of breast cancer patients (*n* = 3554) with low Vs. high expression of HDAC4. Kaplan-Meier survival plot was generated using Kaplan Meier plotter (http://kmplot.com/analysis/), a publicly available tool for meta-analysis based in silico biomarker assessment. This tool uses relapse free survival information downloaded from GEO (Affymetrix microarrays only), EGA and TCGA. The database is handled by a PostgreSQL server, which integrates gene expression and clinical data simultaneously. To analyze the prognostic value of a HDAC4, the patient samples were split into two groups (low vs. high expression of HDAC4). The two patient cohorts were compared by a Kaplan-Meier survival plot, and the hazard ratio with 95 % confidence intervals and logrank P value were calculated. (TIFF 1245 kb)
Additional file 3: Figure S2. HDAC4 expression in ER-positive Vs. ER-negative breast cancer patients, as determined using Oncomine database, a cancer microarray database and web-based data-mining platform. Coexpression analysis was searched with parameters of *p*<0.001 and a fold change >2. The presented data is from Waddell Breast Study [49]. (TIFF 89 kb)

## Abbreviations

CSC: Cancer stem cells
DMSO: Dimethyl sulfoxide
ELISA: Enzyme-linked immunosorbent assay
EMT: Epithelial-mesenchymal transition
ER: Estrogen receptor
HDAC4: Histone deacetylase-4
MCF7TR: Tamoxifen resistant MCF-7 cells
miRNA: microRNA
MTT: 3-(4,5-dimethylthiazol-2-yl)-2,5-diphenyltetrazolium bromide
PBS: Phosphate-buffered saline
PR: Progesterone receptor
RT-PCR: Reverse transcription polymerase chain reaction
TGF-β1: Transforming growth factor-beta 1
