# Supplementary material for: Functional role of miR-10b in tamoxifen resistance of ER-positive breast cancer cells through down-regulation of HDAC4
Source: BMC Cancer. 2015 Jul 24;15:540. doi: 10.1186/s12885-015-1561-x (PMC4512090; doi:10.1186/s12885-015-1561-x)
Supplement: Additional file 1: Table S1. — Oncomine data supporting under-expression of HDAC4 in breast cancer samples, compared to normal controls. (DOCX 25 kb) [file 12885_2015_1561_MOESM1_ESM.docx]

Additional file 1: Table S1 Oncomine data supporting under-expression of HDAC4 in breast cancer samples, compared to normal controls.

| **Dataset / Study** | **Normal Samples** | **Breast Cancer Samples** | **Fold change (HDAC4 expression)^1^** | **P value** |
| --- | --- | --- | --- | --- |
| TCGA Breast | 61 | 389 | -1.636 | 1.40E-19 |
| Curtis [1] | 144 | 1556 | -1.323 | 2.00E-19 |
| Finak [2] | 6 | 53 | -2.516 | 4.66E-4 |
| Radvanyi [3] | 9 | 26 (Ductal) | -2.270 | 0.001 |
|  | 9 | 5 (Lobular) | -6.694 | 0.005 |
| Richardson [4] | 7 | 40 | -2.018 | 0.002 |
| Gluck [5] | 4 | 154 | -1.409 | 0.016 |

**^1^** Fold change in breast cancer samples, relative to normal samples. Negative value represents down-regulation of HDAC4 in breast cancer patients, with significant p values. The data was collected through Oncomine database, a cancer microarray database and web-based data-mining platform.

**References**

1. Curtis C, Shah SP, Chin SF, Turashvili G, Rueda OM, Dunning MJ et al. The genomic and transcriptomic architecture of 2,000 breast tumours reveals novel subgroups. Nature. 2012;486(7403):346-52. doi:10.1038/nature10983.

2. Finak G, Bertos N, Pepin F, Sadekova S, Souleimanova M, Zhao H et al. Stromal gene expression predicts clinical outcome in breast cancer. Nature medicine. 2008;14(5):518-27. doi:10.1038/nm1764.

3. Radvanyi L, Singh-Sandhu D, Gallichan S, Lovitt C, Pedyczak A, Mallo G et al. The gene associated with trichorhinophalangeal syndrome in humans is overexpressed in breast cancer. Proceedings of the National Academy of Sciences of the United States of America. 2005;102(31):11005-10. doi:10.1073/pnas.0500904102.

4. Richardson AL, Wang ZC, De Nicolo A, Lu X, Brown M, Miron A et al. X chromosomal abnormalities in basal-like human breast cancer. Cancer cell. 2006;9(2):121-32. doi:10.1016/j.ccr.2006.01.013.

5. Gluck S, Ross JS, Royce M, McKenna EF, Jr., Perou CM, Avisar E et al. TP53 genomics predict higher clinical and pathologic tumor response in operable early-stage breast cancer treated with docetaxel-capecitabine +/- trastuzumab. Breast cancer research and treatment. 2012;132(3):781-91. doi:10.1007/s10549-011-1412-7.
