# Supplementary figures and images for: Functional role of miR-10b in tamoxifen resistance of ER-positive breast cancer cells through down-regulation of HDAC4
Source: BMC Cancer. 2015 Jul 24;15:540. doi: 10.1186/s12885-015-1561-x (PMC4512090; doi:10.1186/s12885-015-1561-x)

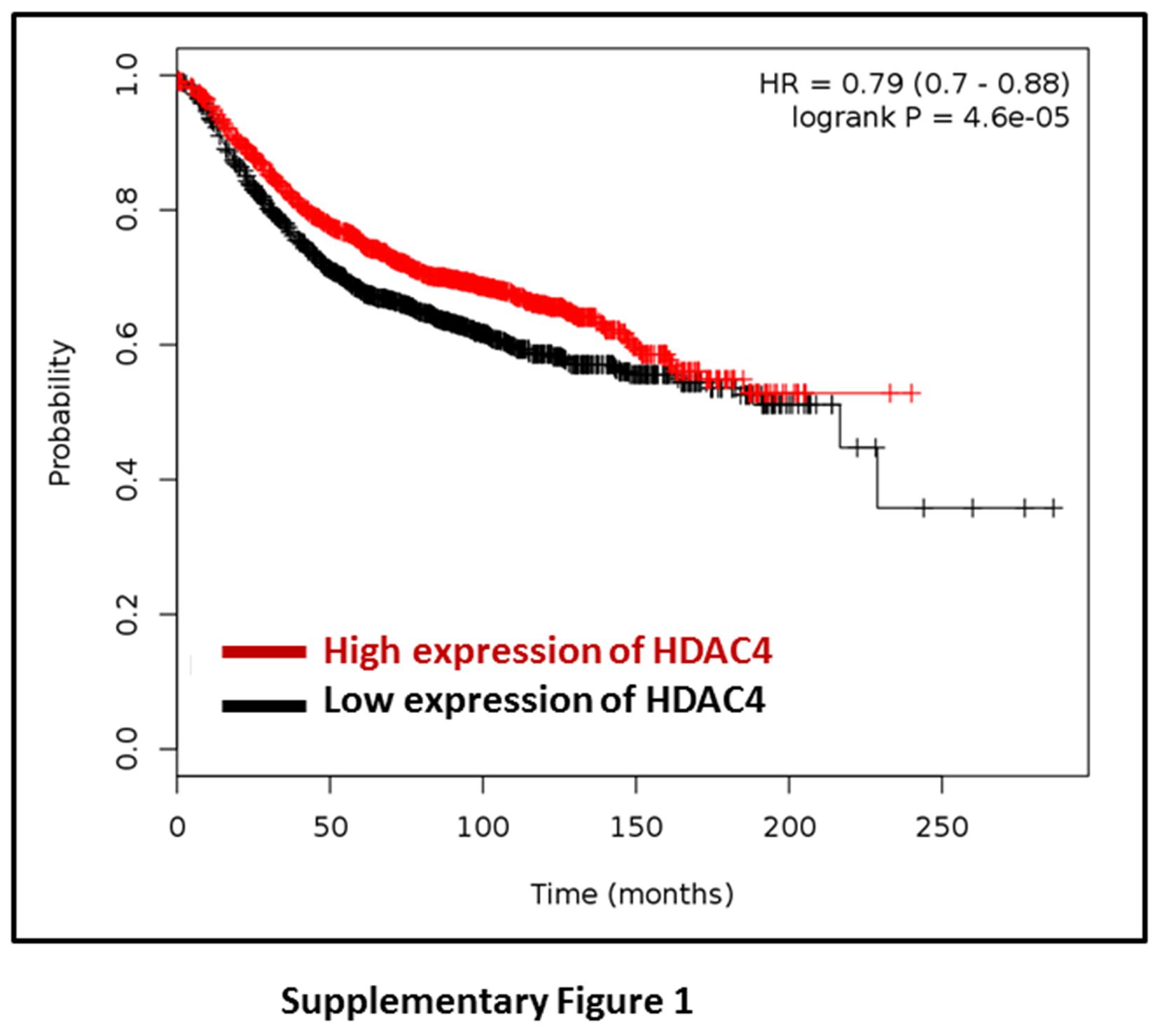

Supplement: Additional file 2: Figure S1. — Comparison of Relapse Free Survival of breast cancer patients (n = 3554) with low Vs. high expression of HDAC4. Kaplan-Meier survival plot was generated using Kaplan Meier plotter (http://kmplot.com/analysis/), a publicly available tool for meta-analysis based in silico biomarker assessment. This tool uses relapse free survival information downloaded from GEO (Affymetrix microarrays only), EGA and TCGA. The database is handled by a PostgreSQL server, which integrates gene expression and clinical data simultaneously. To analyze the prognostic value of a HDAC4, the patient samples were split into two groups (low vs. high expression of HDAC4). The two patient cohorts were compared by a Kaplan-Meier survival plot, and the hazard ratio with 95 % confidence intervals and logrank P value were calculated. (TIFF 1245 kb) [file 12885_2015_1561_MOESM2_ESM.tiff]

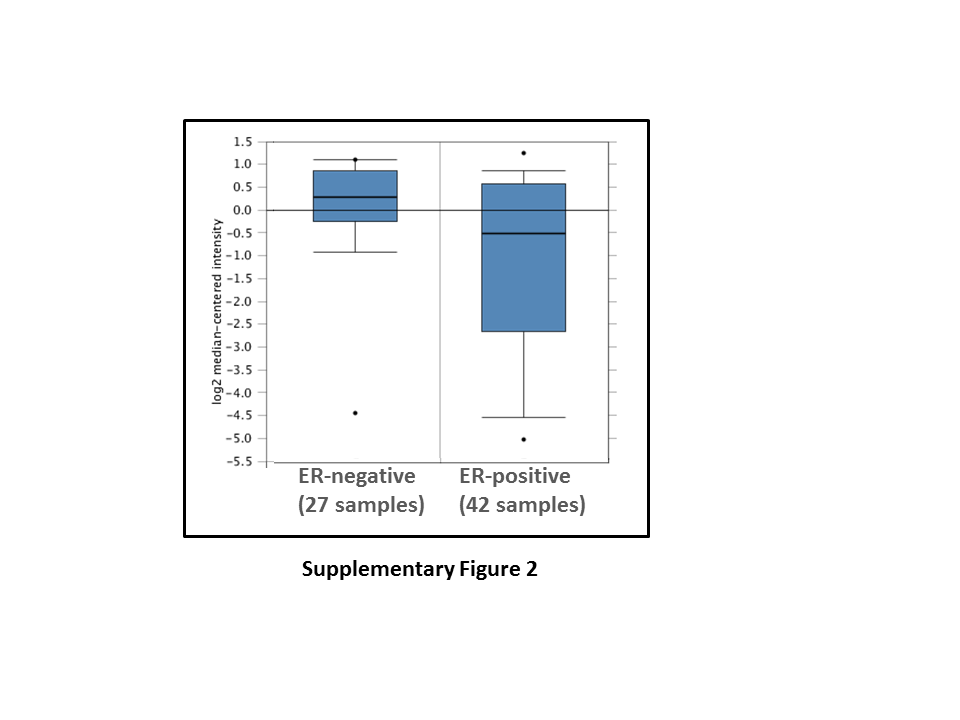

Supplement: Additional file 3: Figure S2. — HDAC4 expression in ER-positive Vs. ER-negative breast cancer patients, as determined using Oncomine database, a cancer microarray database and web-based data-mining platform. Coexpression analysis was searched with parameters of p<0.001 and a fold change >2. The presented data is from Waddell Breast Study [49]. (TIFF 89 kb) [file 12885_2015_1561_MOESM3_ESM.tiff]
